# Pilot Study with regard to the Wound Healing Activity of Protein from *Calotropis procera* (Ait.) R. Br.

**DOI:** 10.1155/2012/294528

**Published:** 2012-08-29

**Authors:** Ramar Perumal Samy, Vincent T. K. Chow

**Affiliations:** Infectious Diseases Programme, Department of Microbiology, Yong Loo Lin School of Medicine, National University of Singapore, Singapore 117597

## Abstract

We provide the scientific basis for the use of *Calotropis procera* for treating skin and wound infections in traditional medicine. The aqueous extract of stem-bark of *C. procera* exhibited more pronounced potent antimicrobial activity. Calo-protein was purified and identified from the most-active aqueous extracts of *C. procera* and showed broad-spectrum activity. Calo-protein inhibited the growth of *S. aureus* and *E. aerogenes* effectively at 25 **μ**g/ml concentration. Mice topically treated with Calo-protein revealed significant wound healing after 14 days comparable to fusidic acid (FA) as positive control. This protein was devoid of cytolytic effect even at higher concentrations on skin cells after 24 h. Further investigation of this Calo-protein of *C. procera* on bacterial inhibition may provide a better understanding of the scientific basis and justification for its use in traditional medicine.

## 1. Introduction

*Staphylococcus aureus* is a leading cause of skin and soft-tissue infections worldwide [1]. *S. aureus* infections are increasingly caused by methicillin-resistant *S. aureus* (MRSA) that has developed resistance to *β*-lactam antibiotics. MRSA is a major problem in healthcare settings [2], with reported incidence rate of invasive MRSA infections of 31.3 per 100,000 individuals, and 20% of these infections resulting in death [3]. World-wide, the rate of methicillin resistance 35.9% is high for surgical [4], chronic (7.4 million) [5], and traumatic wounds (1.5 million). Wound healing is a complex process that involves various inflammatory, proliferative, and remodeling phases [6]. In particular, chronic wounds are major concerns for patient. Since they affect a large number of patients and may reduce their life span [7]. Chronic wounds such as leg ulcers, diabetic foot ulcers, and sores are common in both acute and community healthcare settings [8]. Chronic wounds may be infected by bacteria such as *Streptococcus pyogenes, Enterococcus faecalis, Staphylococcus aureus/MRSA *[9],* Pseudomonas aeruginosa, Enterobacter aerogenes,* and *Escherichia coli, *while fungi and viruses may also cause skin and wound infections [10].

Many bacteria have developed resistance due to the abuse use of antibiotics, while the existing drugs have also caused serious adverse effects in humans [11]. Various preventive and treatment options of wounds are available [8]. However, drugs capable of promoting the wound repair process are still limited. Other considerations include the higher cost for producing synthetic drugs and the various side effects associated with their use [12]. To overcome these issues, the search for alternative agents from plants used in traditional medicine is justified. World-wide, research is conducted to identify new potent, nontoxic wound-healing agents from medicinal plants. Plants are important potential sources of drugs for the biomedical or pharmaceutical industry. Countries such as India and China have rich resources of valuable medicinal plants for the treatment of wound and burns [13]. Approximately 80% of the world population still relies on traditional medicine for the treatment of common diseases [14, 15]. Medicinal plants offer significant potential for the development of novel antibacterial therapies and adjunct treatments [16]. Plant-derived drugs serve as prototypes to develop more effective and less toxic medicines. In previous studies, few attempts were made to confirm the antimicrobial activity of indigenous medicinal plants [17, 18]. Various extracts of medicinal plants were shown to possess antimicrobial activity against *Staphylococcus aureus *[18].

*Calotropis procera* (Ait.) R. Br. is well known for its toxic as well as medicinal properties. The milk weed has been found to be effective in the treatment of leprosy, fever, menorrhagia, malaria, and snake bites. Previous investigation study demonstrated various biological activities of *C. procera* such as anti-inflammatory potential in rats [19, 20], and its osmotin proteins exert antifungal activity [21]. *C. procera* latex administered to rats revealed toxic, wound healing, and pain-killing effects [22]. Chemical compounds in the latex are calotropagenin glycosides/derivatives [23], cardenolides, flavonoids, and saponins [24]. However, the *C. procera* stem bark has hitherto not been well studied completely. Hence, in the present study *C. procera* was evaluated and further characterized for antimicrobial activity and wound-healing potential in mouse model.

## 2. Materials and Methods

### 2.1. Ethnomedicinal Survey

The stem bark of *Calotropis procera* (Ait.) R. Br. (family Asclepiadaceae) plant was collected in May 2004, from the Thiruthani, Tiruvallur district, (near Chennai), Tamil Nadu, India. Plant was identified by a taxonomist with help of Matthew [25]. Voucher specimen was prepared and stored at Entomology Research Institute, India. 

### 2.2. Preparation of Extracts

The stem bark was collected in the field and cleaned by a sterile muslin cloth, cut into small pieces by sterile razor blade, and stored in a sterile polythene bags. All the parts were dried under shade at ambient temperature (31°C) and ground to small powdery granules by electric blender (Preethi, Chennai, India). Using a soxhlet apparatus, the shade-dried and powdered plant material (200 g of each) was extracted with 1000 mL of hexane for 10 h, and successive extracts were done using different organic solvents such as ethyl acetate, dichloromethane, and methanol individually and then finally extracted twice with 600 mL of sterile distilled water (H_2_O) using a shaking water bath at 65°C for 3 h. All the collected extracts were filtered using Whatman number 1 filter paper and evaporated with a rotary evaporator (Buchi, Labortechnik AG, Switzerland) and freeze dryer (lyophilized) to obtain the crude extracts. The dried crude extracts were stored at 4°C for antimicrobial assays [18].

### 2.3. *In Vitro* Antimicrobial Activity

The standard bacterial cultures of Gram-negative (*Escherichia coli, Enterobacter aerogenes, Proteus vulgaris,* and *Proteus mirabilis)* and Gram-positive (*Pseudomonas aeruginosa* and *Staphylococcus aureus) *bacterial strains were obtained from the Department of Microbiology, National University of Singapore, Singapore. The strains were stored at −70°C, subcultured on 20 mL Mueller Hinton (MH) agar plates (pH 7.4), and incubated overnight at 37°C prior to use. The antimicrobial property was tested using the disc-diffusion method [26]. Five young colonies of each strain of bacteria taken from their respective cultures grown overnight on MH agar plates (Oxoids, UK) were suspended in 5 mL of sterile saline (0.9%), and the density of the suspension adjusted to approximately 3 × 10^8^ colony forming unit (CFU). The swab was used to inoculate the dried surface of MH agar plate by streaking four times over the surface of the agar and rotating the plate approximately 90°C to ensure an even distribution of the inoculums. The medium was allowed to dry for about 3 min before adding a 6 mm diameter sterile paper disc (Beckton Dickson, USA) on the surface. Each disc was tapped gently down onto the agar to provide a uniform contact. 100 *μ*g/mL of each plant extract (lyophilized aqueous residues) was weighed and dissolved in 1 mL of water, and 20 *μ*L of the extracts (containing 100 *μ*g of residues) were applied on each disc (3 replicates). The sterile blank disc served as a normal control. The antimicrobial activity of the extracts on the clinical isolates was determined in comparison with the reference antibiotic (chloramphenicol 30 *μ*g/disc), which was used as a positive control. The plates were incubated at 37°C for 24 h, and the inhibition zones measured and calculated. 

### 2.4. Phytochemical Screening of Extracts

The phytochemical screening was done on the extracts using the chemical method previously reported for the detection of secondary metabolites [27]. 

### 2.5. Extraction and Isolation

The most active aqueous extract of stem bark of *C. procera* was used for the purification of antimicrobial agents [27]. The residue 0.5 gm was dissolved completely in 5 mL of 50 mM Tris-HCl (pH 7.4), after centrifugation at 12,000 rpm for 15 min, and clear solution was filtered by a membrane filter (0.22 *μ*m). Sample 1 mL diluted (1 : 5 ratio) in 4 mL of Tris-HCl and injected into a Superdex G-75 column linked to high-performance liquid chromatography (HPLC AKTA, Denmark) resolved five fractions (P1–P5). Fraction (1 mL) was monitored at 280 nm and collected. All the fractions were screened against bacteria, of which fraction (P2) exerted higher antimicrobial activity. The most active fraction (P2) was further separated by C18 reverse-phase (RP)-HPLC column, which gave four fractions (CF-1–CF-4). Resultant separation of active fraction (CF-1) displayed maximum inhibitory effect against bacteria that was eluted by C8 RP-HPLC column, final yields of the fraction purity were determined by matrix-assisted laser desorption ionization-time of flight/mass (MALDI)-TIF/MS and designated as “Calo-protein.” The concentrated protein was then tested (100 *μ*g/mL) against bacteria *in vitro*.

### 2.6. Animals

Eight-week-old male Swiss Albino mice, body weight ranging from 25–30 gm, were obtained from the NUS animal breeding center Sembawang, Singapore. All animals were kept in individual cages (Darzon, Laboratory, USA) with standard laboratory conditions with dark/light cycle (24 ± 2.0), food pellet and water ad libitum used in wound healing model of experiments.

#### 2.6.1. Mice Model of Wound

Mice were divided into three groups, each group consists of three mice each (*n* = 3), respectively, (IACUC Protocol number 691/04). The dorsal skin of the mice was marked by permanent marker and then cleaned with 70% ethyl alcohol (Merck, Germany) for surface sterilization before shaved by sterile surgical blade [28]. The animals were anesthetized (75 mg/kg of ketamine + 0.1 mg/kg of medetomidine) and full thickness of 16 mm (8 × 8 mm) for excision wound created and 50 *μ*L of bacteria (1 × 10^5^ colony forming units per mL). Group I infected mice wound received only saline served as a control, Group II infected mice wound treated with 5 mg/kg, body weight of Calo-protein mixed with aqueous cream (ICM Pharma Pte Ltd., Kallang Place, Singapore) formulations were topically applied on the dorsal side of the mice, Group III infected mice applied 5 mg/kg, b.w of fusidic acid (FA) sodium salts (Sigma Co., St. Louis, USA) served as an antibiotic control. The wounds were left open without any dress and kept individually in separate cages. The wound area monitored and measured every day up to 14 days (2-week time). 

#### 2.6.2. Measurement of Wound Area

Treatment with Calo-protein and standard drug were performed by topical application on the wound surface up to 4 days. The wound areas were traced on millimeter in diameter (mm^2^) by tracer paper (Merck Co., Germany) immediately after the wound created and every day until the healing was completed. The percentage of wound reduction in the wound size was calculated according to the following formula:

(1) $$\begin{array}{c} \text{Wound}\;\text{contraction}\;\left(\%\right)=\frac{W_{c}-W_{t}}{W_{c}}\times 100, \end{array}$$

where *W*_*c*_ is the wound area immediately after wound creation as a control (*c*), *W*_*t*_ is the wound area on day of treatment (*t*).

#### 2.6.3. Histological Evaluation

The experiment was terminated after 14 days and the wound removed by surgically for histopathological examination. The tissues were cut and trimmed down and processed in dehydration in alcohol series 50–90% for 30 min each, 100% ethyl alcohol (I/II), and histoclear 100% (I) for 60 min each, and 100% histoclear (II) for overnight. The decalcification was done with molted wax at 55°C for 1 h in each jar. The blocks were prepared by using wax, 5 *μ*m thick sections were stained with haematoxylin and eosin (H&E) and imaged by Olympus Light microscope (Olympus America Inc., PA, USA).

#### 2.6.4. Measurement of Collagen

Healed tissues of the wounds were cut, weighed (100 mg), and homogenized with 1 mL of T-PER buffer (Thermo Scientific, IL, USA). The homogenates were centrifuged at 10,000 rpm for 10 min at 4°C. The supernatant collected and collagen content in the Calo-protein, FA-treated, and wound control mice were determined by ELISA using mouse antitype I collagen assay kit (Chondrex, Inc., Redmond, WA, USA) as per the manufactures instructions.

### 2.7. Cytotoxicity Assay

The cytotoxic potential of different crude extracts of *C. procera *was evaluated against human macrophage (U-937) cell lines by XTT assay at 2000, 1000, 500, 250, 100, 50, 25, and 12.5 *μ*g/mL [29]. The cell proliferation assayed in 96-well microtitre plates, confluent cells (5 × 10^6^ cells per well) were incubated with diverse extracts for 24 h and the inhibitory concentrations recorded. Further, cytotoxic and cytolytic effects of purified protein tested on human skin fibroblast cells (HEPK) were evaluated by measuring the release of lactate dehydrogenase (LDH) enzyme using a cytotoxicity detection kit (Roche Mannheim, Germany) at various concentrations (1000–0.001 *μ*g/mL). After 24 h, 200 *μ*L of aliquot of the cell supernatant obtained from each 96-well plate was used for the quantification of cell death and cell lysis, based on the measurement (450 nm). LDH activity was released from the cytosol of damaged cells into the supernatant. The assay was performed in triplicate (*n* = 3), percentage cell proliferation and cytolytic effects were calculated.

### 2.8. Statistical Analysis

The data presented as mean ± standard deviation (SD), each concentration tested three replicates (*n* = 3), inhibitory concentrations millimeter (mm) in diameter. The percentage reduction of wound data was compared using one-way analysis of variance (ANOVA) followed by Dunnett's tests. Values with *P* less than *P > 0.01, and **P > 0.05 compared with the control group were considered as statistical significance.

## 3. Results 

### 3.1. Ethnobotanical Survey and Application in Healthcare

**Figure 1:**
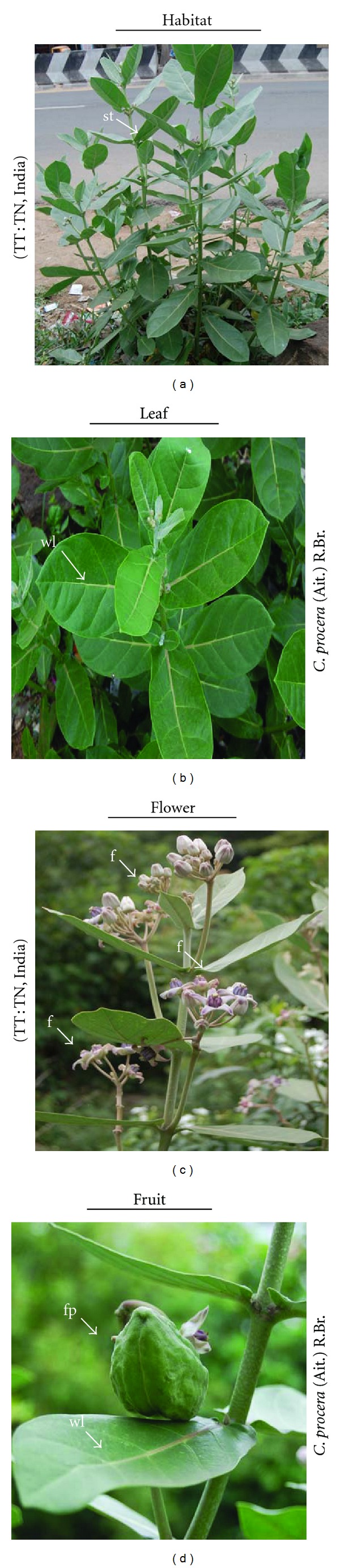
The giant milkweed *Calotropis procera *growing as a spreading shrub or small tree has a simple stem with only few branches. (a, b) Large, dark-green leaves in opposite pairs along smooth stem. (c) Beautiful waxy white flowers have deep purple spots or blotches at the base of each five petals. (d) It produces a fleshy fruit with inflated pod containing several brown seeds with white long silky hair. It exudes a milky white latex when cut or broken. St: stem, f: flower, fp: fruit pod, and wl: waxy leaf surface.

This study attempts to provide the scientific basis for the traditional beliefs of this herbal remedy. We surveyed the traditional medical knowledge on *C. procera *from the local practitioners for preparing home remedies for their routine healthcare (Figures 1(a), 1(b), 1(c), and 1(d)). In Tamil Nadu, decoction of the stem bark is traditionally used for the treatment of skin diseases, while the aerial parts of *C. procera* are used for treating fever, pain, muscular spasm, and so forth. The milky latex wet with a clean cloth was applied mainly on affected areas of cut wounds, thorn injuries, and inflamed swellings. The botanical/family name, yield of extracts, and their activities are provided.

### 3.2. *In Vitro* Antimicrobial Activity

Table 1 shows the *in vitro* antimicrobial, cytotoxic, and phytochemical properties of organic and aqueous extracts of stem bark of *C. procera* as a traditional medicinal plant. Among the tested extracts, ethyl acetate, methanol, and aqueous extracts displayed high antimicrobial activity against the tested bacteria. The methanol and aqueous extracts revealed relatively broad-spectrum activity against bacteria at 100 *μ*g/mL concentration. Aqueous extracts exhibited more pronounced antimicrobial activity with the largest inhibitory zones of 28 mm diameter compared to the standard chloramphenicol antibiotic. Overall, methanol and aqueous extracts of *C. procera *exerted a broad activity against both Gram-negative and Gram-positive bacteria. However, the hexane and dichloromethane extracts exhibited weaker activity against the tested bacteria, while the hexane, ethyl acetate, and dichloromethane extracts did not exhibit any effect against *E. coli* and *P. aeruginosa*.

**Figure 2:**
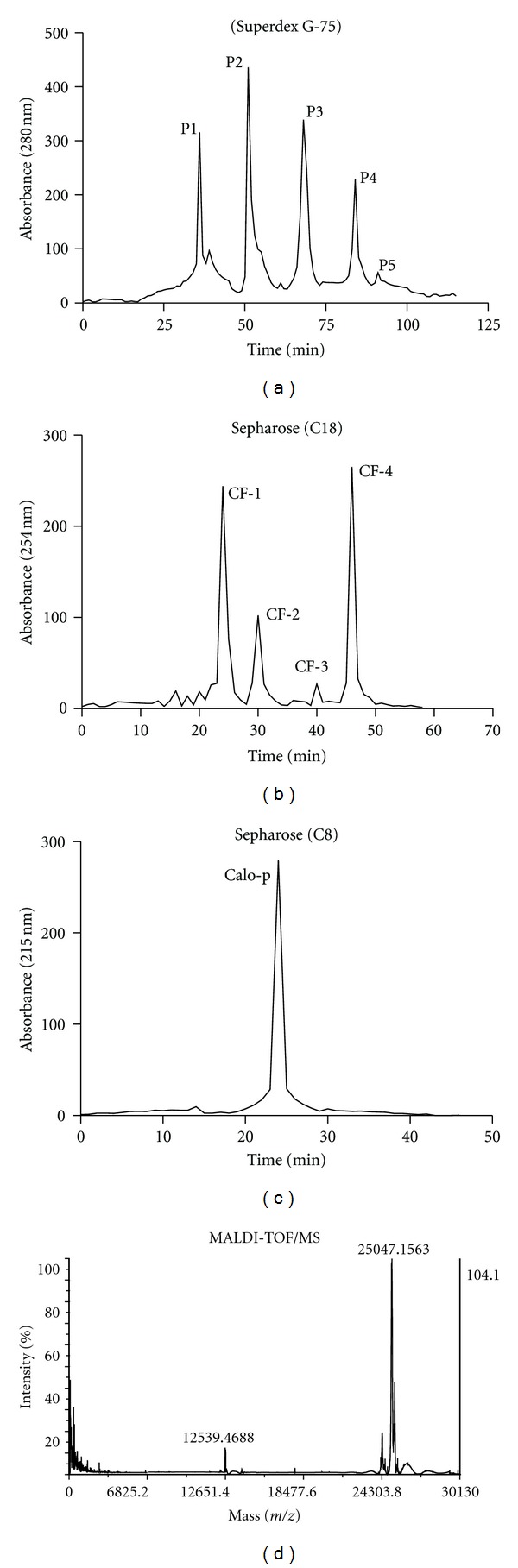
(a) The most active stem bark extract was separated by Superdex G-75 column and resolved into five fractions (P1–P5). (b) The active fraction (P2) was further eluted and gave fractions CF-1–CF-F4. (c) The final pure fraction was separated by reverse-phase HPLC column (C8) using CF-1 fraction and designated as Calo-protein (Clo-p) of *C. procera. *Calo-p was tested for *in vitro *antimicrobial efficacy at various dose and in wound healing studies in mouse model. (d) The molecular mass of the Calo-protein was determined by MALDI-TOF/MS.

**Figure 3:**
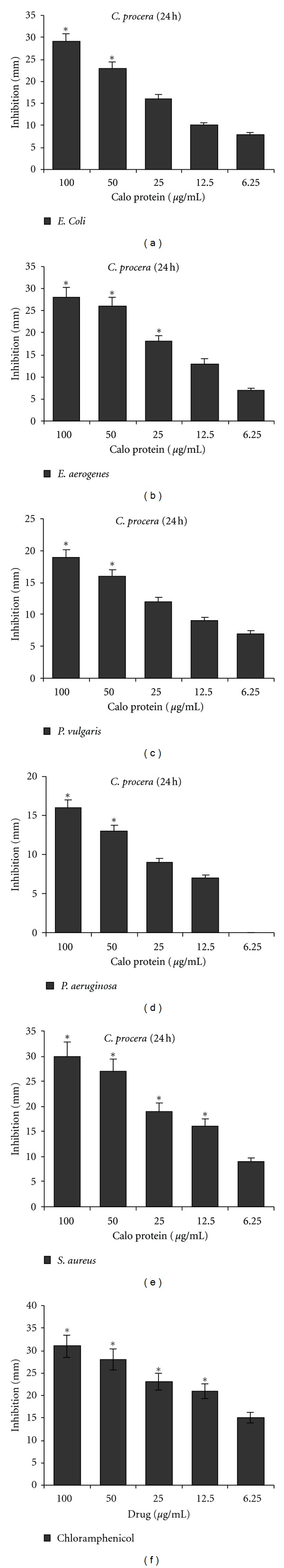
Antimicrobial properties of purified protein from the most active *C. procera *extract tested against bacteria that cause skin infections. (a–f) Results are expressed as the mean ± SD (*n* = 3) of the inhibition zones (diameter around the discs). The original size of disc was 6 millimeter in diameter. The bacterial growth inhibitory efficacy was compared with chloramphenicol used as a positive control.

The most active aqueous crude extract of *C. procera* was purified by superdex G-75 (Figure 2(a)), reverse-phase HPLC columns C18 (Figure 2(b)). The final fraction was designated as Calo-protein (Figure 2(c)), the purity of its molecular weight of the isolated Calo-protein determined by MALDI-TOF/MS (Figure 2(d)). The Calo-protein was tested against bacteria at a wide range of concentrations (100–6.25 *μ*g/mL). The purified Calo-protein showed broad-spectrum activity against tested bacteria (Figure 2(c)). However, the Calo-protein inhibited the growth of *S. aureus *and* E. aerogenes* effectively at 25 *μ*g/mL concentrations. It showed largest inhibitory zones (30 mm) equal to chloramphenicol (31 mm). Even at the lowest concentration of Calo-protein, the growth of *S. aureus, E. aerogenes, P. vulgaris, P. mirabilis, *and* E. coli* was effectively inhibited. *P. aeruginosa* was weakly inhibited by Calo-protein at all the tested concentrations. Finally, the purified protein from the *C. procera* stem bark exerted the strongest antimicrobial activity against *S. aureus* than the crude extracts (Figure 3).

### 3.3. Phytochemical Screening

Phytochemical screening of the stem bark of *C. procera* indicated the presence of various secondary metabolites such as alkaloids, flavonoids, tannins, coumarins, anthraquinones, saponins, cardiac glycosides, sterols, and teriterpenes. The crude extracts of this plant are relatively rich in glycosides, alkaloids, tannins, sterols, and terpenes which may inhibit the growth of organisms.

### 3.4. Cytotoxicity of Crude Extracts

The hexane, ethyl acetate, and dichloromethane crude extracts of *C. procera* showed toxicity to human macrophages (U-937) to 250 and 500 *μ*g/mL, However, methanol and aqueous extracts were less toxic up to >2000 *μ*g/mL, whereas the lower concentrations (100–12.5 *μ*g/mL) were devoid of toxic effects and morphological changes of cells (Table 1). However, varied toxic effects were observed in the *C. procera* crude extracts in a dose-dependent manner compared to control cells.

### 3.5. Cytotoxic Effect of Protein

**Figure 4:**
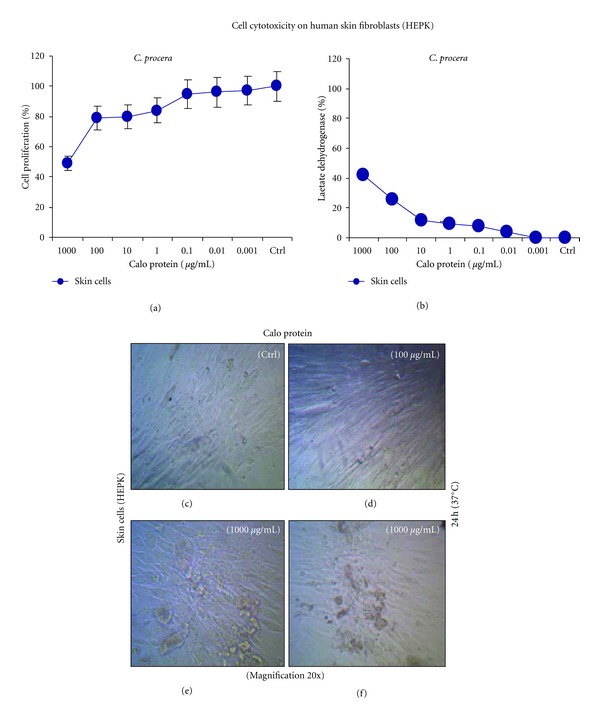
Cytotoxic effect of Calo-protein from the stem bark of *C. procera *assayed on human skin cells and their morphological changes observed for 24 h. (a) Calo-protein was tested against skin cell up to 1000 *μ*g/mL concentrations for 24 h. There were no toxic effects recorded at high concentrations. (b) The protein did not produce severe cytolytic effects after 24 h exposure of the plant protein *in vitro*. Light microscopic images showing the morphological changes of human skin fibroblast cells were exposed to the Calo-protein obtained from the stem bark of *C. procera *tested at varying doses (1000–0.001 *μ*g/mL). (c) There was no alteration in the control cells, (d) while lower dose (100 *μ*g/mL) of protein did not affect the cell morphology (e, f), the higher dose of protein (1000 *μ*g/mL) showed some changes on HEPK cells after 24 h. Untreated cells served as a control (ctrl).

Normal human skin fibroblast (HEPK) cells were exposed to Calo-protein to assay for cytotoxicity varying concentrations. However, this protein did not exert any toxic effect on skin cells even at higher concentrations (1000 *μ*g/mL) (Figure 4(a)). Furthermore, the Calo-protein did not display any cytolytic effects at all the tested concentrations after 24 h compared with control cells (Figure 4(b)). The purified bioactive protein of *C. procera* did not show toxic effects on skin cells up to 1000 *μ*g/mL. Light microscopic images of human skin fibroblasts were exposed to the Calo-protein at varying doses (1000–0.001 *μ*g/mL). There was no alteration in the control cells (Figure 4(c)). Whereas the lower dose (100 *μ*g/mL) of protein did not affect the cell morphology (Figure 4(d)), but the higher dose of protein (1000 *μ*g/mL) showed some changes on HEPK cells after 24 h (Figures 4(e) and 4(f)).

### 3.6. Wound Healing Effect of Calo-Protein

**Figure 5:**
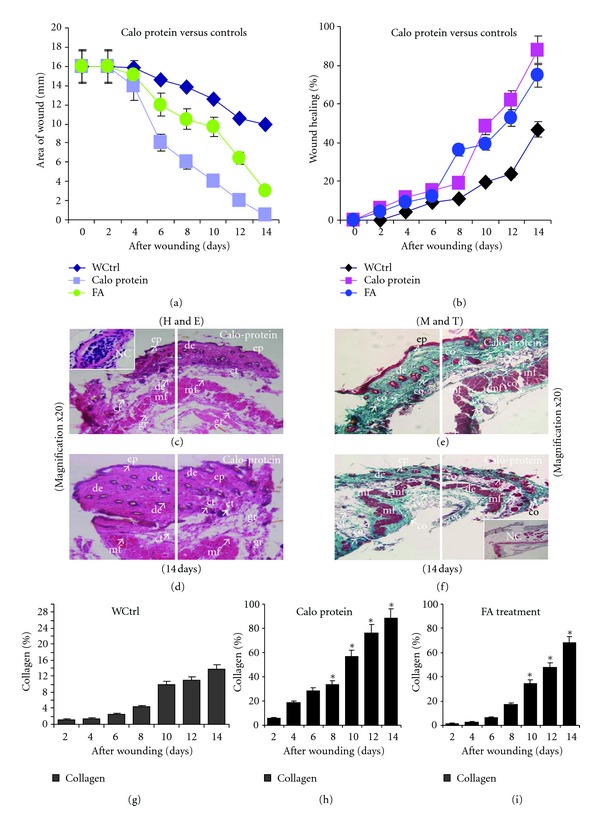
Wound-healing activity by *C. procera *protein was determined in a mouse model. (a) Reduction of wound area was compared with antibiotic (FA) up to 14 days. (b) Percentage of wound healing effect of Calo-protein was compared with the control group. (c, d) The protein treatment showed full thickness of reepithelialization in the epidermis, well-organized granular layer, compared to the wound control. (e, f) Masson's Trichrome (MT) staining showed less deposition of collagen in the wound control mice after 14 days. (g–i) Biochemical analysis showed that protein treated enhanced more collagen synthesis than the FA and WCtrl after 14 days. ep: epidermis, de: dermis, ct: cortex, ne: neutrophils, mf: muscle fiber, and co: collagen.

Significant wound healing activity was recorded in the mice treated with Calo-protein after 14 days compared to control (Figures 5(a) and 5(b)). There was a considerable reduction in wound area from day 4 onwards in treated mice compared to mice receiving fusidic acid. The treatment with Calo-protein accelerated the rate of wound closure or repair much faster than the control mice. In the histopathological examination, there was notably less noticeable infiltration of inflammatory cells, greatly increased blood vessel formation, and proliferation of cells by the Calo-protein. Interestingly, there was full thickness of reepithelialization in the epidermis, well-organized granular layer, compared to the control. The wounds of animal treated with Calo-protein showed full-thickness of epidermal regeneration that covered completely the wounded area. The epidermis was thick and disorganized especially compared with the adjacent normal skin and complete epithelialization, vascularization, and hair follicles formation were observed in treated mice (Figures 5(c), 5(d), 5(e), and 5(f)). This protein exerted a positive impact on wound healing by enhanced cellular proliferation, granular tissue formation and epithelialization, and early dermal and epidermal regeneration. In addition, topical application of the Calo-protein of mice pronounced in more collagen content than the FA-treated mice versus control mice (Figures 5(g), 5(h), and 5(i)).

## 4. Discussion

Our findings confirmed the antimicrobial property of *C. procera* used in traditionally medicine for the treatment of skin and wound-related disorders. We previously reported the antimicrobial activity [30] of certain traditional medicinal plants used in Tamil Nadu, India. Of these, the aqueous extracts of *C. procera, *exhibited showed the most significant antimicrobial effect against *S. aureus *and *E. aerogenes*. Interestingly, the growth of several wound causing bacterial was controlled by organic and aqueous extracts. Out of 5 different extracts that were examined, 3 extracts were failed to show any antimicrobial effect against *E. coli *and *P. aeruginosa *at the tested concentrations. This negative effect may be attributed to the different climatic and edaphic factors influencing the secondary metabolites. Another study reported the lack of antimicrobial compounds inadequate concentrations in the extracts [31].

Both the organic and aqueous extracts showed antimicrobial activity against wound-causing bacteria. Notably, the aqueous extract of *C. procera* exerted comparatively stronger effect against *S. aureus, E. aerogenes,* and *P. mirabilis*. In addition, the inhibitory activities of *C. procera *extracts were comparable to that of the reference antibiotic. Previously, it was reported that the apical twigs and latex of *C. procera* produced greatest inhibition zones against *S. aureus *[32]. Furthermore, we demonstrated the high antimicrobial potency of the *C. procera *extract against all the tested bacteria may be due to the high content of glycosides and various proteins present in the aqueous extracts [23, 24]. The differences in the antimicrobial activity of various extracts may be directly related to the diversity of compounds accumulated in *C. procera*: proteins [21], calotropagenin glycosides [23], cardenolides, flavonoids, and saponins [24] thus corroborating our results. Such compounds can bind the Gram-negative bacteria to form a heavy soluble complex on the cell surface that subsequently disturbs the interaction between bacteria and cell receptors. 

Previously, Freitas et al. [33] studied the enzymatic activities and protein profile of latex of the *C. procera*. The *C. procera* protein showed broad-spectrum activity against tested bacteria at 100–6.25 *μ*g/mL concentrations. We showed that the Calo-protein inhibited the growth of *S. aureus *and* E. aerogenes,* and it was equivalent to chloramphenicol. Even at the lowest dose of protein, the growth of *S. aureus, E. aerogenes, P. vulgaris, P. mirabilis, *and* E. coli* was inhibited effectively. The ethyl acetate and dichloromethane crude extracts of *C. procera* showed toxicity to human macrophage (U-937) cells at 250 and 500 *μ*g/mL doses than the methanol and aqueous extracts. However, the pure protein failed to show toxic effects on human skin (HEPK) cells at doses of up to 1000 *μ*g/mL. Magalhães et al. [34] studied that the various organic extracts of the stem of *C. procera* and found that ethyl acetate and acetone extracts displayed higher cytotoxic potential against tumor cells (IC 0.8–4.4 mL), but the methanolic extract was weakly cytotoxic. Similarly, laticifer proteins (LPs) obtained from the latex of *C. procera *displayed considerable cytotoxic effects (IC 0.42–1.36 *μ*g/mL) on SF295 and MDA-MB435 cells. In addition, LP was shown to inhibit DNA synthesis and to target DNA topoisomerase I triggering apoptosis in cancer cells [35].

**Wound Healing Effect of Calo-Protein**

The latex of *C. procera* has been used in traditional medicine to treat different inflammatory diseases. The anti-inflammatory activity of latex proteins has been well documented using different inflammatory models [36]. Wound healing events involve different phases such as preventing blood loss, inflammation, epithelial repair such as proliferation [7], mobilization, migration, differentiation, tissue remodeling, and collagen deposition. The acute inflammatory phase involved in wound healing is accompanied by the synthesis of extracellular matrix that is remodeled to the formation of scar tissue, connective tissue, collagenization, and acquisition of wound strength [37]. Our mouse model revealed enhanced rate of wound contraction than the control. The mice treated with 5 mg/kg Calo-protein showed favorable results compared with other groups. The treated wound exhibited marked dryness of wound margins with tissue regeneration at 14 days. The healing potential of *C. procera *on dermal wounds in guinea pigs has been documented [37]. In this study, the topical application of Calo-protein constituent accelerated wound repair, with full-thickness coverage of the wound area by organized epidermis. The wound healing potential of Calo-protein is corroborated by documented anti-inflammatory effect of the *C. procera *plant in rats [19, 20]. The Calo-protein led to potent wound contraction comparable with FA treatment. In addition, it had favorable effects on inflammation and wound healing.

Histological examination showed that increased cellular infiltration in treated cases may be due to the chemotactic effect enhanced by the extract that attracts inflammatory cells towards the wound site. Augmented cellular proliferation may be due to the mitogenic activity of the Calo-protein that may have contributed significantly to the repair process. Dermal regeneration in treated mice also implied that the protein exerted a positive impact on cellular proliferation, granular tissue formation and epithelization. The final stage of the healing process involves collagen deposition and remodeling within the dermis [38]. Thus, the MT staining clearly supported the wound repair process in Calo-protein-treated mice that showed more pronounced collagen accumulation than the control. Several types of secondary metabolites and active compounds isolated from plants have been documented in animal models as the active principles for influencing wound healing. The latex of *C. gigantean* was previously shown to have wound healing effects in rats comparable to those of nitrofurazone, and the extract contains high content of glycosides, flavonoids, phenolic, and triterpenoid compounds with antimicrobial and antioxidant properties [28]. In our study, Calo-protein isolated from the aqueous extract of *C. procera* possesses wound-healing property against bacterial infections. It was previously reported that calactin, mudarin, and calotropain are active constituents of *C. procera* latex [39], with antimicrobial and antioxidant properties.

## 5. Conclusions

The therapeutic potency of *C. procera* demonstrated that the wound-healing activity of this protein was effective for inhibition of bacterial pathogens. We conclude that the Calo-protein from the *C. procera* stem bark has potential to be developed into new therapeutic agent(s) for wound healing against bacterial infections.

## Figures and Tables

**Table 1:** Antimicrobial activity of different extracts of stem-bark of *C. procera *tested on Gram-positive and Gram-negative bacteria at 100 *μ*g/mL concentration and their cytotoxic potential was evaluated against human macrophage (U-937) cell lines by XTT assay at 2000, 1000, 500, 250, 100, 50, 25, and 12.5 *μ*g/mL doses.

Crude extracts	Phytochemical screening	Yield	*E. coli *	*E. aerogenes*	*P. vulgaris *	*P. mirabilis *	*P. aeruginosa *	*S. aureus *	Cytotoxicity (*μ*g/mL)
(1) Hexane	Flovanoids	5.36 gm	—	11 ± 0.94	7 ± 0.1	9 ± 0.06	—	8 ± 0.12	<250
(2) Ethyl acetate	Steroids	3.42 gm	—	18 ± 0.06	17 ± 0.27	15 ± 0.2	—	25 ± 0.06	<500
(3) Dichloromethane	Polyphenols	4.78 gm	—	12 ± 0.4	8 ± 0.23	11 ± 0.48	—	13 ± 0.21	<500
(4) Methanol	Terpenoids	5.98 gm	14 ± 0.31	19 ± 0.2	23 ± 0.4	20 ± 0.6	8 ± 0.12	27 ± 0.06	>2000
(5) Aqueous	Glycosides; Tannins; saponins	7.15 gm	11 ± 0.14	22 ± 0.7	25 ± 1.3	20 ± 0.12	9 ± 0.23	28 ± 0.15	>2000
(6) Chloramphenicol	Standard antibiotic	30 *μ*g	21 ± 0.34	24 ± 0.3	23 ± 0.12	26 ± 0.23	10 ± 0.35	29 ± 0.12	—

Clinical isolates of Gram-positive and Gram-negative bacteria were used. Values are mean ± SD of three replicates (*n* = 3), inhibition zones in diameters (mm) produced by the extract around the discs. Size of disc was 6 mm. Absence of bacterial inhibition denoted by (—), that is, no inhibition zone around the disc by crude extract of plants.

## Abbreviations

Calo-p: *Ca* *lo* *tr* *op* *is* *procera* protein
XTT: (2,3-Bis-(2-methoxy-4-nitro-5-sulfophenyl)-2H-tetrazolium-5-carboxanilide)
FA: Fusidic acid (sodium salts)
MRSA: Methicillin resistant *Staphylococcus* *aureus*
MH: Mueller Hinton agar
CFU: Colony forming unit
CHL: Chloramphenicol
HPLC: High-performance liquid chromatography
CF: *Ca* *lo* *tr* *op* *is* *procera* fraction
*W*_*c*_: Wound control
*W*_*t*_: Wound treatment
T-PER: Tissue protein extraction reagent
ELISA: Enzyme-linked immunoabsorbance assay
U-937: Human macrophage
HEPK: Human skin fibroblast
LDH: Lactate dehydrogenase enzyme
SBE: Stem-bark extract
LP: Latex proteins.

## References

[1] Frei CR, Makos BR, Daniels KR, Oramasionwu CU (2010). Emergence of community-acquired methicillin-resistant *Staphylococcus aureus* skin and soft tissue infections as a common cause of hospitalization in United States children. *Journal of Pediatric Surgery*.

[2] Pichereau S, Rose WE (2010). Invasive community-associated MRSA infections: epidemiology and antimicrobial management. *Expert Opinion on Pharmacotherapy*.

[3] Klevens RM, Morrison MA, Nadle J (2007). Invasive methicillin-resistant Staphylococcus aureus infections in the United States. *Journal of the American Medical Association*.

[4] Moet GJ, Jones RN, Biedenbach DJ, Stilwell MG, Fritsche TR (2007). Contemporary causes of skin and soft tissue infections in North America, Latin America, and Europe: report from the SENTRY Antimicrobial Surveillance Program (1998–2004). *Diagnostic Microbiology and Infectious Disease*.

[5] Driscoll P Wound prevalence and wound management, in clinical practice, market data, medtech, surgery, wound management.

[6] Li J, Chen J, Kirsner R (2007). Pathophysiology of acute wound healing. *Clinics in Dermatology*.

[7] Bowler PG, Duerden BI, Armstrong DG (2001). Wound microbiology and associated approaches to wound management. *Clinical Microbiology Reviews*.

[8] Cullum N, Nelson EA, Flemming K, Sheldon T (2001). Systematic reviews of wound care management: (5) beds, (6) compression, (7) laser therapy, therapeutic ultrasound, electrotherapy and electromagnetic therapy. *Health Technology Assessment Journal*.

[9] Kučišec-Tepeš N, Bejuk D, Košuta D (2006). Characteristics of war wound infection. *Acta Medica Croatica*.

[10] Frank C, Bayoumi I, Westendorp C (2005). Approach to infected skin ulcers. *Canadian Family Physician*.

[11] Blaser M (2011). Antibiotic overuse: stop the killing of beneficial bacteria. *Nature*.

[12] MacDonald J, Asiedu K (2010). WAWLC: world alliance for wound and lymphedema care. *Wounds*.

[13] Kumar B, Vijayakumar M, Govindarajan R, Pushpangadan P (2007). Ethnopharmacological approaches to wound healing-exploring medicinal plants of India. *Journal of Ethnopharmacology*.

[14] de Smet PAGM (2002). Herbal remedies. *New England Journal of Medicine*.

[15] Poonam K, Singh GS (2009). Ethnobotanical study of medicinal plants used by the Taungya community in Terai Arc Landscape, India. *Journal of Ethnopharmacology*.

[16] Mahady GB (2005). Medicinal plants for the prevention and treatment of bacterial infections. *Current Pharmaceutical Design*.

[17] Engel LW, Straus SE (2002). Development of therapeutics: opportunities within complementary and alternative medicine. *Nature Reviews Drug Discovery*.

[18] Jeevan Ram A, Bhakshu LM, Venkata Raju RR (2004). *In vitro* antimicrobial activity of certain medicinal plants from Eastern Ghats, India, used for skin diseases. *Journal of Ethnopharmacology*.

[19] Kumar VL, Chaudhary P, Ramos MV, Mohan M, Matos MPV (2011). Protective effect of proteins derived from the *Latex of Calotropis procera* against inflammatory hyperalgesia in monoarthritic rats. *Phytotherapy Research*.

[20] Tour N, Talele G (2011). Anti-inflammatory and gastromucosal protective effects of *Calotropis procera* (Asclepiadaceae) stem bark. *Journal of Natural Medicines*.

[21] de Freitas CDT, Lopes JL, Beltramini LM, de Oliveira RS, Oliveira JT, Ramos MV (2011). Osmotin from Calotropis procera latex: new insights into structure and antifungal properties. *Biochimica et Biophysica Acta*.

[22] Thankamma L (2003). Hevea latex as a wound healer and pain killer. *Current Science*.

[23] Kanojiya S, Madhusudanan KP (2012). Rapid Identification of calotropagenin glycosides using high-performance liquid chromatography electrospray ionisation tandem mass spectrometry. *Phytochemical Analysis*.

[24] Moustafa AMY, Ahmed SH, Nabil ZI, Hussein AA, Omran MA (2010). Extraction and phytochemical investigation of Calotropis procera: effect of plant extracts on the activity of diverse muscles. *Pharmaceutical Biology*.

[25] Matthew KM (1981–1983). *The Flora of Tamil Nadu Carnatic*.

[26] Bauer AW, Kirby WM, Sherris JC, Turck M (1966). Antibiotic susceptibility testing by a standardized single disk method.. *American Journal of Clinical Pathology*.

[27] Harborne JB (1976). *Phytochemical Methods*.

[28] Saratha V, Subramanian S, Sivakumar S (2010). Evaluation of wound healing potential of calotropis igantea latex studied on excision wounds in experimental rats. *Medicinal Chemistry Research*.

[29] Elsinghorst EA (1994). Measurement of invasion by gentamicin resistance. *Methods in Enzymology*.

[30] Samy RP, Ignacimuthu S (2000). Antibacterial activity of some folklore medicinal plants used by tribals in Western Ghats of India. *Journal of Ethnopharmacology*.

[31] Ahmad I, Beg AZ (2001). Antimicrobial and phytochemical studies on 45 Indian medicinal plants against multi-drug resistant human pathogens. *Journal of Ethnopharmacology*.

[32] Parabia FM, Kothari IL, Parabia MH (2008). Antibacterial activity of solvent fractions of crude water decoction of apical twigs and latex of *Calotropis procera* (Ait.) R. Br. *Natural Product Radiance*.

[33] Freitas CDT, Oliveira JS, Miranda MRA (2007). Enzymatic activities and protein profile of latex from Calotropis procera. *Plant Physiology and Biochemistry*.

[34] Magalhães HIF, Ferreira PMP, Moura ES (2010). *In vitro* and *in vivo* antiproliferative activity of calotropis procera stem extracts. *Anais Of Academia Brasileira de Ciências*.

[35] Soares de Oliveira J, Pereira Bezerra D, Teixeira de Freitas CD (2007). *In vitro* cytotoxicity against different human cancer cell lines of laticifer proteins of *Calotropis procera* (Ait.) R. Br. *Toxicology in Vitro*.

[36] Lima-Filho JV, Patriota JM, Silva AFB (2010). Proteins from latex of *Calotropis procera* prevent septic shock due to lethal infection by *Salmonella enterica serovar Typhimurium*. *Journal of Ethnopharmacology*.

[37] Rasik AM, Raghubir R, Gupta A (1999). Healing potential of *Calotropis procera* on dermal wounds in Guinea pigs. *Journal of Ethnopharmacology*.

[38] Hernandez V, Recio MDC, Manez S, Prieto JM, Giner RM, Rios JL (2001). A mechanistic approach to the *in vivo* anti-inflammatory activity of sesquiterpenoid compounds isolated from *Inula viscose*. *Planta Medica*.

[39] Parrotta JA (2001). *Healing Plants of Peninsular India*.

